# Mental health burden of high school students, and suggestions for psychosocial support, 1.5 years into the COVID-19 pandemic in Austria

**DOI:** 10.1007/s00787-022-02032-4

**Published:** 2022-07-28

**Authors:** Rachel Dale, Andrea Jesser, Christoph Pieh, Teresa O’Rourke, Thomas Probst, Elke Humer

**Affiliations:** Department for Psychosomatic Medicine and Psychotherapy, University for Continuing Education Krems, Krems an der Donau, Austria

**Keywords:** Adolescents, Mental health, COVID-19, Psychological support

## Abstract

The COVID-19 pandemic and containment efforts seem to be particularly challenging for adolescents. This study assessed mental health in high school students 1.5 years after the pandemic began in Austria. A cross-sectional survey was carried out from September to November 2021. Well-being (WHO-5), depressive symptoms, anxiety symptoms and sleep quality were assessed. A total of 1505 adolescents participated (78% girls). The cut-offs for clinically relevant symptoms were exceeded by 62% girls and 38.1% boys for depression (PHQ-9 score ≥ 11), 49% girls and 29% boys for anxiety (GAD-7 score ≥ 11) and 28% girls and 17% boys for insomnia (ISI score ≥ 15). The prevalence of suicidal ideation (item 9 of the PHQ-9) within the last 2 weeks was 47% in girls and 32% in boys. These data collected in autumn 2021 (the start of the second semester of reopened schools; t2) were compared to data collected in February 2021 (one semester after remote schooling; t1). A matched pairs analysis according to age, gender, region, school type and migration background resulted in a total sample size of *N* = 2514 adolescents. Results showed small deteriorations in mental health (i.e., well-being, depression, insomnia, suicidal ideation) in girls at t2 compared to t1, and an increase in suicidal thoughts in boys (all *p*-values < 0.05). Qualitative data show that young people have a need for more psychological support, both professional and informal, as well as increased mental health literacy. Results suggest that mental health burden in adolescents remains high 1.5 years into the pandemic and highlight the need to implement timely psychological support.

**Supplementary Information:**

The online version contains supplementary material available at 10.1007/s00787-022-02032-4.

## Introduction

It is becoming apparent that the COVID-19 pandemic is long-term in nature, proving a sustained challenge to mental health. Although some studies show some improvement again in mental health of the general population on average since the initial decline in early 2020 [1, 2], many studies show that the pandemic has not been uniform in its effects [3] and there are some vulnerable groups [2, 4] which do still warrant special attention. Young people have been particularly affected psychologically [5–9]. This has been explained by the specific biographical challenges and biopsychosocial changes of adolescence [5]. Young people increasingly detach themselves from their parents and the nuclear family. In the context of important decisions for their future life path, autonomy, self-regulation and self-determination gain importance. New developmental environments and interaction with peers play an integral role in this process. Reorientation to peers of the same age facilitates young people's development into independent adults, allowing them to develop a sense of social self-identity while building stronger bonds with their peer group [10]. As an essential context for peer interaction and the acquisition of knowledge and personal maturity, school contributes significantly to the development of adolescent identity and interpersonal relationships [11].

During the COVID-19 pandemic, students were repeatedly taught via distance learning over a longer period of time. School closures and curfews resulted in the loss of critical social contacts with classmates and friends. Furthermore, the introduction of distance learning changed daily routines overnight [12]. Adolescents found themselves at home with highly stressed parents working from home [13], which exacerbated conflicts between parents and children [14]. In addition to fears about their health and that of relatives and friends, research revealed that adolescents increasingly worry about their professional future and the long-term socioeconomic consequences of the pandemic [15]. These may be some of the reasons for the high psychological stress among young people. Given that many mental disorders begin in childhood or adolescence and this age group is more susceptible to long-term consequences of mental health [16], it is essential to continue to assess their mental health during this prolonged global crisis.

Each country has had their own unique experience of the pandemic (in terms of restrictions, deaths, school closures, etc.) so it is important to provide data from as many countries as possible. Furthermore, results can help guide country-specific public health policy related to mental health. In line with this, we previously assessed the mental health of adolescents in Austria in February 2021, after one semester of remote schooling and during a time where several national lockdown measures were in place [17]. These findings indicated that mental well-being and life satisfaction were significantly impaired 1 year after the beginning of the COVID-19 pandemic in Austria compared to pre-pandemic [18]. Another survey was conducted in June/July 2021, when there were no lockdown restrictions and schools had been re-opened for one semester. Although mental health symptoms remained poor in comparison to the available pre-pandemic data, a small improvement was observed in June/July compared to February [19]. In line with this, Riiser et al. [20] found that well-being depended on the magnitude of restrictions and children reported the challenges of distance learning and school as an important place for socializing as well as learning ([21], but see [22]).

These findings led to some cautious optimism that school openings and easing of other restrictions may have led to an improvement in the psychological well-being of high school students [23]. However, it is important to monitor the robustness of these improvements and continue to monitor the situation as the long-term nature of the pandemic becomes more apparent. Furthermore, although some improvements may be expected, we did not predict a return to pre-pandemic levels of mental health. As such it is also important to consider solutions and investigate what young people need at this time.

The aim of the current study was to quantitatively assess the mental health status of Austrian adolescents in autumn 2021. During this time in Austria the schools were fully open and there was no lockdown, however, the rates of infection were starting to rise again. A further aim was to compare the quantitative, validated questionnaires providing objective measures of mental health statistically with a matched sub-sample from the survey conducted in February 2021 to assess the progression of mental health symptoms over the course of the pandemic. Additionally, we aimed to put this information in context by qualitatively investigating potentially effective and concrete strategies to assist the Austrian youth in their mental well-being during this challenging time.

## Methods

A cross-sectional online survey was conducted via REDCap [24] from 14th September 2021 to 14th November 2021. School representatives posted the link to the online survey via social media platforms to students and invited them to participate. The survey was conducted at the beginning of the second semester of reopened schools (see supplementary materials for more details). At the time of the survey in Austria schools were fully open and there was no lockdown in place. Protective measures in classes included regular COVID-19 tests and wearing face masks outside the classroom. General COVID-19 measures in Austria during the time of the survey relied mainly on presenting proof of vaccination, recovery from COVID-19 or a negative test to visit restaurants, sports centers, take part in events etc.

To assess the progression of mental health symptoms over the course of the pandemic quantitatively, the sample collected from September to November 2021 (t2) was matched via propensity score matching with a sub-sample from the survey conducted in February 2021 (t1). Details from this survey have been published elsewhere [17]. In brief, high school mainly took place in remote schooling from October 2020 until the 8th February 2021 and a national lockdown was in place. After this some aspects of the lockdown were lifted and schools re-opened but in a shift system, with students sometimes attending in presence and sometimes from home.

### Measures

The measures were chosen as standard and broad measures of mental health which would have comparability with other COVID-19 studies [4], particularly in Austria [9, 25], and with pre-COVID-19 data [26]. We limited the measures to the following to avoid the survey becoming too long.

*Well-being:* well-being was measured using the World Health Organisation-five well-being index (WHO-5; [27], containing five questions with total scores ranging from 0 (no well-being) to 100 (maximal well-being).

*Depression:* Depression symptoms were assessed using the patient health questionnaire-9 (PHQ-9; [28]). The nine items ask about the last two weeks and yield a total score ranging from 0 to 27. The cut-off for clinically relevant symptoms is ≥ 11 for adolescents [29].

*Suicidal ideation:* Item 9 of the PHQ-9 asks: “Over the last two weeks, how often have you been bothered by thoughts that you would be better off dead or of hurting yourself in some way?”. Response to this question was coded in a binary way to detect any recent suicidal ideas within the last two weeks (presence of suicidal thoughts = response to item 9 ranged from 1 to 3; absence of suicidal thoughts = response to item 9 was 0).

*Anxiety*: The generalised anxiety disorder (GAD-7) scale was used to measure anxiety [30]. The seven items have a maximum score of 21 and the cut-off for clinically relevant anxiety symptoms is ≥ 11 in adolescents [31].

*Sleep:* Insomnia and sleep quality were assessed with the insomnia severity index (ISI; [32]). The seven-item scale has a maximum score of 28 and a cut-off of ≥ 15 for moderate insomnia [33].

*Gender* was coded as girl, boy or non-binary. Each gender group was analysed separately because (a) girls are over-represented in the sample, thus biasing the results of the full sample, and (b) it is known that there are gender differences in mental health scores [6].

*Migration background:* To assess the migration status, students were asked whether they and/or both parents were born abroad.

*Support:* At the end of the survey students were asked whether they would want support to improve their psychological wellbeing? Students who affirmed this question, were asked about the type of support they would like to have (“Please briefly describe what kind of support would be helpful for you”). In a comment box, they could respond to this question freely without character limit.

More details on the measures can be found in the supplementary materials.

### Analyses

#### Quantitative analyses

Descriptive statistics, chi-squared tests, univariate as well as multivariate analysis of variance were computed to analyse the data from the survey conducted from 14th September 2021 to 14th November 2021 (t2).

Respondents to the survey conducted at t2 were matched with respondents from the survey conducted from 3rd February 2021 to 28th February 2021 (t1) using propensity score matching [34]. A propensity score is assigned to each participant, representing the probability of belonging to one of the two groups, given a vector of observed covariates; in this case age, gender, region, migration background and school type. This score is then included as a covariate in all future analyses [35]. Multiple matching techniques were assessed via jitter plots, histograms and percent balance improvement and ‘nearest neighbour’ was selected as having the best matching.

After matching the two groups (t1 and t2) were compared, with the mental health measures as the dependent variables and time point and propensity score as fixed factors, using linear models for continuous measures and general linear models with the binomial family for assessing the likelihood of being over the cut-off score.

Effect sizes are shown as Cohens *d*. *p*-values were 2-tailed, and statistical significance was set at *p* = 0.05.

#### Qualitative analyses

We analysed open answers from t2 on support services using a conventional approach to qualitative content analysis [36]. We received 393 free text comments. Most comments were brief (one word up to one sentence), but some of them also comprised more detailed descriptions. To familiarise ourselves with the material and obtain a sense of the whole, we first read all the comments. In a next step, we assigned initial codes to the responses and in this process grouped similar codes into overarching categories. After initial coding of the whole data set, we went through the material a second time and checked the allocations of comments and the sharpness of the categories. To facilitate the coding process, we used Microsoft Excel. One researcher performed the coding and grouping. Interim results were discussed and revised with another researcher to corroborate findings.

## Results

A total of 1505 adolescents participated in the survey in autumn 2021 (t2), and 1173 (77.9%) were female. Their mean (SD) age was 16.3 (1.4) years, and 249 (16.5%) had a migration background.

### Quantitative results

Measures of self-reported psychological health of the whole t2 sample by gender are summarized in supplementary Table S1. All variables were significantly affected by gender at t2 (all *p* < 0.0001), with worst scores in non-binary students. Girls showed worse mental health compared to boys. See the supplementary materials for more details on the full t2 sample. The following results discuss the matched sample.

Of 1505 high school students participating at t2, 1257 could be matched to participants from t1, leading to a total sample size of N = 2514. The matched sample at t1 (*N* = 1257) comprised 77.2% (*n* = 971) girls, 20.4% (*n* = 256) boys and 2.4% (*n* = 30) non-binary adolescents with a mean age of 16.3 (SD = 1.38) years and 16.6% (*n* = 209) had a migration background. The matched sample at t2 (*N* = 1257) comprised 78% (*n* = 981) girls, 19.3% (*n* = 242) boys and 2.7% (*n* = 34) non-binary adolescents, with a mean age of 16.3 (SD = 1.4) years and 16.6% (*n* = 209) had a migration background.

Table 1 summarizes the results of the mental health measurements and cut-off values by gender and time point. Table 2 shows the statistical results. Matched-sample analyses showed a mean (SD) change from time 1 to time 2 for girls’ self-ratings of well-being [WHO-5: 37.7 (20.1) to 34.82 (20.2)], depressive symptoms [PHQ-9: 11.6 (6.43) to 13.0 (6.32)], and insomnia [ISI: 10.2 (5.47) to 10.8 (5.7); all *p* < 0.05]. For boys and adolescents with non-binary gender, no differences in mean scores were observed between t1 and t2. At t2 the number of girls exceeding the cut-off for clinically relevant depression (61.8%) and insomnia (26.4%) increased compared to t1 (53.2% depression and 21.0% insomnia; all *p* < 0.01). The prevalence of suicidal ideation was higher in girls (47.2%) and boys (33.5%) at t2 as compared to t1 (girls: 35.3%, boys: 30.6%) with *p* < 0.0001. Effect sizes for the significant findings were small, with a range from 0.05 to 0.23 (Table 2). Although girls had worsened mean scores and increased prevalences for most variables investigated at t2 compared with t1, only one significant difference (suicidal ideation) was observed for boys and no significant differences were observed for adolescents with non-binary gender.

**Table 1:** Measures of psychological health by gender and time point (matched sample)

		t1				t2			
		Total	Girls	Boys	Non-binary	Total	Girls	Boys	Non-binary
WHO-5	*N*	1257	971	256	30	1257	981	242	34
	Score, mean (SD)	38.4 (21.0)	37.7 (20.1)	42.8 (23.7)	22.5 (16.0)	36.5 (21.2)	34.8 (20.2)	45.3 (23.1)	23.9 (18.0)
PHQ-9	*N*	1180	935	219	26	1257	981	242	34
	Score, mean (SD)	11.3 (6.65)	11.6 (6.43)	9.44 (6.98)	18.1 (5.51)	12.5 (6.62)	13.0 (6.32)	9.67 (6.84)	18.9 (5.69)
	≥ 11, No. (%)	606 (51.4)	497 (53.2)	85 (38.8)	24 (92.3)	731 (58.2)	606 (61.8)	93 (38.4)	32 (94.1)
	Suicidal ideation, No. (%)	419 (33.3)	330 (35.3)	67 (30.6)	22 (84.6)	575 (45.7)	463 (47.2)	81 (33.5)	31 (91.)
GAD-7	*N*	1205	959	220	26	1257	981	242	34
	Score, mean (SD)	10.1 (5.34)	10.5 (5.12)	8.23 (5.74)	13.7 (5.24)	10.1 (5.37)	10.6 (5.19)	7.62 (5.42)	12.9 (4.81)
	≥ 11, No. (%)	542 (45.0)	450 (46.9)	74 (33.6)	18 (69.2)	579 (46.1)	484 (49.3)	71 (29.3)	24 (70.6)
ISI	*N*	1244	971	245	28	1257	981	242	34
	Score, mean (SD)	9.84 (5.6)	10.2 (5.47)	8.24 (5.86)	11.9 (5.01)	10.5 (5.83)	10.8 (5.7)	8.76 (5.87)	13.9 (6.19)
	≥ 15, No. (%)	247 (19.9)	204 (21.0)	37 (15.1)	6 (21.4)	316 (25.1)	259 (26.4)	42 (17.4)	15 (44.12)

*N* sample size, *SD* standard deviation, *t1* 1 semester after almost exclusively remote schooling (February 3rd to February 28th 2021), *t2* at the beginning of the second semester of reopened schools (14th September 2021 to 14th November 2021), *ISI* insomnia severity index, *GAD-7* generalized anxiety disorder 7 scale, *PHQ-9* patient health questionnaire 9 scale, *WHO-5* well-being questionnaire of the World Health Organization (WHO)

**Table 2:** Statistics for the comparison between t1 and t2 (matched sample analyses)

		Girls		Boys		Non-binary	
		Statistic [CI]	*p*-value	Statistic [CI]	*p*-value	Statistic [CI]	*p*-value
WHO-5	Mean score	*t*_1949_ = − 3.13 [− 4.65, − 1.07] *d* = 0.07 [− 0.11, − 0.03]	< 0.01	*t*_495_ = 1.19 [− 1.63, 6.62]	0.24	*t*_61_ = 0.3 [− 7.27, 9.87]	0.76
PHQ-9	Mean score	*t*_1913_ = 4.85 [0.84, 1.98] *d* = 0.11 [0.07, 0.15]	< 0.0001	*t*_458_ = 0.36 [− 1.04, 1.5]	0.72	*t*_57_ = 0.52 [− 2.16, 3.69]	0.6
	Cut-off ≥ 11	*Z*_1913_ = 3.82 [0.17, 0.54] *d* = − 0.18 [0.09, 0.27]	< *0*.001	*Z*_460_ = − 0.08 [− 0.39, 0.36]	*0.94*	*Z*_59_ = 0.29 [− 1.88, 2.48]	0.78
	Suicidal ideation	*t* = 5.09 [0.28, 0.64] *d* = 0.23 [0.14, 0.32]	< 0.0001	*t* = 0.94 [− 0.2, 0.57] *d* = 0.09 [− 0.1, 0.29]	< 0.0001	*t* = 1.24 [− 0.34, 1.55] *d* = 0.3 [− 0.17, 0.78]	0.21
GAD-7	Mean score	*t*_1937_ = 0.42 [− 0.36, 0.56]	0.67	*t*_459_ = − 1.17 [− 1.63, 0.41]	0.24	*t*_57_ = − 0.62 [− 3.31, 1.74]	0.54
	Cut-off ≥ 11	*Z*_1939_ = 1.06 [− 0.08, 0.27]	0.29	*Z*_461_ = − 1.02 [− 0.6, 0.19]	0.31	*Z*_59_ = 0.18 [− 1.05, 1.25]	0.86
ISI	Mean score	*t*_1949_ = 2.42 [0.12, 1.11] *d* = 0.05 [0.01, 0.10]	< 0.05	*t*_484_ = 0.98 [− 0.53, 1.57]	0.33	*t*_59_ = 1.37 [− 0.92, 4.9]	0.18
	Cut-off ≥ 15	*Z*_1951_ = 2.8 [0.09, 0.51] *d* = 0.15 [0.04, 0.25]	< 0.01	*Z*_486_ = 0.68 [− 0.32, 0.65]	0.5	*Z*_61_ = 1.84 [− 0.04, 2.26] *d* = 0.53 [− 0.02, 1.13]	0.07

*p*-values (2-tailed), *t1* 1 semester after almost exclusively remote schooling (February 3rd to February 28th 2021), *t2* at the beginning of the second semester of reopened schools (14th September 2021 to 14th November 2021), *ISI* insomnia severity index, *GAD-7* generalized anxiety disorder 7 scale, *PHQ-9* patient health questionnaire 9 scale, *WHO-5* well-being questionnaire of the World Health Organization (WHO), Cohen’s *d* was calculated as an effect size measure for differences (small effect: 0.2–0.5, medium effect: 0.5–0.8, large effect: > 0.8). *CI* 95% confidence interval

### Qualitative results

**Fig. 1:**
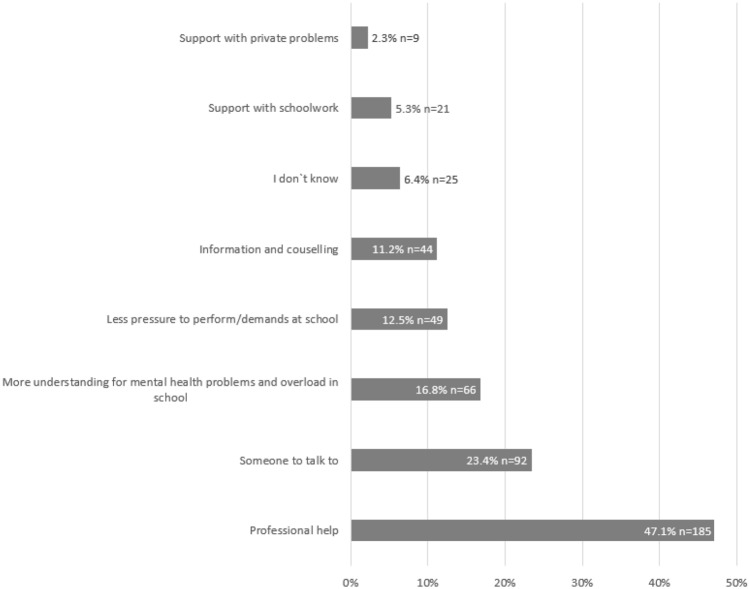
Sources of support. The percentages of students reporting each main category of response that emerged from the open-ended question “What type of support would be helpful for you?”

At the end of the survey, students were asked whether they wish for support to improve their psychological well-being, which was affirmed by 37.2% (*N* = 557) of all respondents. Of the 557 students who stated that they would like to have support, 393 gave more detailed information about the type of support. These results broken down by gender can be found in the supplementary materials. Qualitative data analysis resulted in eight categories (Fig. 1). The category “professional help” was endorsed by *n* = 185 (47.1%), referring to psychological support, psychotherapy, medication, or outpatient and inpatient psychiatric help. Students mentioned the need for more free or affordable services and less waiting time. Especially school psychologists would often only be available to a very limited extent. In addition, they addressed the need for more low-threshold services in the context of school, as these can be more easily integrated into their daily routines. Students also added that, on the one hand, they would like to have anonymous (online) services that parents do not have to be informed about, and on the other hand, they would like to have compulsory services that provide the impetus when the inhibition threshold is too high to seek help themselves. They would like professional help for themselves, but also for others, if they notice that their friends and classmates need support.

Therapy, only that there is no therapist nearby who is less than 1 h away by public transport and who has a place on a health insurance scheme. And my mother doesn't have enough money to pay for it herself. (non-binary student no. 1425, age 15).

At school, a school psychologist who really does her job and is not just never there for 3/4 of the school year or at times when there are no students. Most of the time, getting help from a psychological point of view fails because you have no idea where to start and don't want to tell 1000 people that you have a problem. (female student no. 117, age 18).

Further *n* = 93 (23.7%) stated that someone to talk would provide helpful support. In this category, students referred to 1. outside third parties with or without expertise, but not in the context of professional services, 2. family and friends, and 3. guidance teachers (Vertrauenslehrer). The young people expressed the need for someone who listens, is understanding and unbiased, does not judge and with whom they can talk confidentially. It is clear from their statements that what is important is to address fears and insecurities, to receive positive affirmations and to strengthen their self-worth.

It might be a bit unrealistic, but I would like to find some kind of "subtle" help. Something that is not labelled as “psychotherapy” or anything like that. Sometimes I would like to ask for help, but I am afraid that I will be treated differently by friends and family. I don't want that carefree, happy way of interacting to be replaced with pity. I am also afraid that this would make me less credible or worth taking seriously by others. (male student no. 1751, age 18).

More understanding for mental health problems and overload in school also appeared as a topic (*n* = 66, 16.8%). Students stated that teachers, but also parents, have little understanding for the fact that school performance cannot be achieved to the required extent due to mental health problems or obligations outside of school. They experience little flexibility in adapting requirements to individual situations. They perceive learning goals to be rigid and feel that efforts are not sufficiently recognised. Students also criticised that they have little opportunity to have a say in school decisions and do not feel that their concerns are heard.

Further 12.5% (*n* = 49) mentioned less pressure to perform and/or fewer demands at school would help. This category includes statements that school requirements are stressful and that students would like COVID-19 related alleviations, e.g., reduction of subject matter, cancellation of the oral school-leaving exam.

Information and counselling was stated by 11.2% (*n* = 44). Students mentioned that they would like more information and counselling on mental illness: what it is, what to do about it, how many young people are affected by it, how to help others, etc. Young people suggested that counselling and information could take place in the context of school: in special classes, workshops or information days that could be organised by external lecturers. They suggested that it would be important to raise awareness among young people, but also among teachers and parents, about the needs of young people and to destigmatise mental illness. They also stated that it would be helpful to learn in the exchange with the group/class that others also have problems and to get the necessary information about what could help oneself or friends (help for self-help) or when one needs professional help.

For me, education in schools would be important. If all my classmates were aware of how important mental health is, but also how common mental illnesses are, I think it would make a lot of things easier. However, education of adults is almost more lacking. I don't know how the federal school representatives or anyone else could enforce this, but I think it is important to be aware that one's own child is also exposed to mental stress, even if perhaps nothing was done wrong in the upbringing. (female student no. 26, age 14).

For more example quotes, see the supplementary materials. ‘I don't know’ was the response of *n* = 25 (6.4%). This category includes statements in which students either explicitly or implicitly express that they are feeling bad but do not know or cannot articulate what would be helpful. This may be because they do not know what help is available or because known services such as psychotherapy or school psychology are too stigmatised.

Support with schoolwork (*n* = 21; 5.3%) and support with private problems (*n* = 9; 2.3%) also emerged. The first category includes statements such as tutoring for individual school subjects, support with time management or with organising and scheduling school assignments. The second category refers to statements in which students express the need for help with private or family problems, but without specifying more precisely what kind of support this could be.

## Discussion

The findings of this study suggest that from February 2021 to autumn 2021 girls’ mental health has worsened in the areas of well-being, depression, suicidal thoughts, and sleep. Although little change has occurred in boys’ mental health during this time, suicidal thoughts have increased significantly. Likewise, non-binary adolescents showed similar scores in early and late 2021. The lack of change in non-binary youth’s mental health may, however, be due to a ceiling effect as their scores were already very poor in the first survey. In addition, the non-binary and male samples were smaller than the female sample, and significant results are more probable in larger samples.

At this point in the pandemic, it appears that the mental health of high school students is not directly linked to school openings or lockdowns. Furthermore, as new data emerge, it seems possible that factors other than school and restrictions may also be having detrimental effects on youth mental health. Özlü-Erkilic et al. [37] investigated 15–25-year-olds in Turkey and Austria at two time points (May–June 2020 and September–October 2020) and found a significant decrease in mental health between the two time points. As the pandemic progressed, the estimated severity of COVID-19 infection, the fear of the individual and/or a family member being infected and ruminations about COVID-19 were rated higher across all groups. Therefore, rather than demonstrating resilience and coping, young people seem to be experiencing deteriorating mental health as the pandemic continues.

Similarly in adults, compared to during the first lockdown, mental health did not improve soon after the lockdown or six months after the end of the first lockdown in Austria [38, 39]. Furthermore, an Austrian study of 12 waves between April and December 2020 found depressive symptoms accumulated over time, worsening with each lockdown but not improving in the periods of reduced restrictions in between, and this was particularly the case for young people (16–29 years; [40]).

As such, it seems that although school closures and restrictions likely play a role in mental health [19, 20], more general pandemic factors are also involved, such as rises in daily COVID cases [37], slow vaccination rates in Austria, exhaustion/depletion of mental resources [41], and concerns about the future [4]. These are in addition to the non-pandemic mental health challenges already faced by adolescents, particularly girls [42, 43]. Further studies are required to better understand the underlying reasons for the persistent mental health burden in adolescents.

Given the sustained psychological burden experienced by young people, we also aimed to investigate potential intervention strategies, as suggested by the youth themselves. In terms of improving the youth mental health situation, responses from the children suggest a great demand for providing opportunities to talk about their problems. Considering that the onset of most mental disorders usually occurs earlier in life [16], it seems of utmost importance to initiate effective support at an early stage. Professional help was requested by almost half (47%) of those who answered the question. This can be explained by the fact that despite years of efforts to expand psychosocial services in Austria [18], there are still gaps in the care infrastructure, both in diagnostics and in therapy [44]. For example, there is still a shortage of child and adolescent psychiatrists [45, 46]—there is one specialist in child and adolescent psychiatry for every 30,000 adolescents [47] and there are long waiting times for psychotherapy as a statutory health insurance benefit [48]. With around 1.1 million students, 181 school psychologists are available at the schools to advise not only the students but also teachers, parents and school supervisors [49]. The effects of the pandemic have further exacerbated the shortage of services, which is why there is an urgent need to expand services and provide free counselling and therapy for children and adolescents.

In addition to professional support, simply having someone to talk to, such as peers or counsellors, was also mentioned relatively often (24%). It is possible that the high number of mentions in this category reflects the fact that mental illness is still associated with a stigma, which, in addition to feelings of shame, self-blame or self-harm, can lead to young people not seeking professional help [50–52]. Many young people confide in their peers and friends rather than in adult caregivers and outside counsellors [53]. Peer relationships protect against psychological problems or help to cope with them better [54]. Subjective well-being is also higher among those who are less likely to use social withdrawal as a coping strategy. Given the relevance of confidential communication for coping with stress and psychological problems, it is important to encourage and support young people to seek low-threshold support from peers and to accept help on a personal as well as on a professional level. In Austria, there is at least one promising initiative; the online platform https://open2chat.at/ was developed on an evidence-based approach and connects young people from the age of 14 with trained people from their age group via chat in order to talk about their problems.

Other implementable strategies requested by respondents included wishing for more information regarding mental health problems via advice, explanations and integration in the curriculum. This could help to improve mental health literacy [55] by 1. enabling young people to recognise and classify their symptoms of psychological distress, 2. providing them with concrete tools to help themselves and others or to assess at which point additional external help is needed, 3. improving their knowledge about where to seek help for mental health problems, 4. creating a space for exchange where problems can be discussed and classmates can get to know each other better and 5. reducing the stigma of being affected by mental health problems and seeking help for them [56, 57]. To address our respondents request, our department, in collaboration with other experts, has developed a website aimed at young people to explain various mental health issues and provide tips for improving mental health (www.istokay.at).

There are some important limitations to note. The cross-sectional nature means that causal effects of the pandemic from t1 to t2 cannot be concluded. Although self-rating instruments can be implemented remotely and reach a larger sample, the use of clinical interviews would be optimal., Lastly there is the possibility of a self-selection bias due to the online implementation of the study.

Overall, these results highlight the continuing psychological struggles of young people and as such the need to implement timely mental health promotion and prevention strategies to mitigate the mental burden in young people caused by the COVID-19 pandemic and associated measures.

## Supplementary Information

Below is the link to the electronic supplementary material.

Supplementary file1 (DOCX 89 KB)

## Data Availability

The raw data supporting the conclusion of this article will be made available by the authors upon reasonable request.
