# Supplementary material for: Mental health burden of high school students, and suggestions for psychosocial support, 1.5 years into the COVID-19 pandemic in Austria
Source: Eur Child Adolesc Psychiatry. 2022 Jul 28;32(6):1015–24. doi: 10.1007/s00787-022-02032-4 (PMC9330952; doi:10.1007/s00787-022-02032-4)
Supplement: Supplementary file 1 — Supplementary file1 (DOCX 89 KB) [file 787_2022_2032_MOESM1_ESM.docx]

Mental health burden of high school students, and suggestions for psychosocial support, 1.5 years into the COVID-19 pandemic in Austria

Supplementary Materials

Methods

Measures:

Well-being (WHO-5): This is a reliable and validated 5-item questionnaire using a 6-point likert scale [1]. Cronbach’s alpha for the current t2 sample = 0.86

Depression (PHQ-9): The PHQ-9 has been validated for adolescents [2]. It consists of nine self-report items on four-point scales ranging from 0 to 3, with a maximal total score of 27. Cronbach’s alpha for the current t2 sample = 0.88.

Anxiety (GAD-7): The GAD-7 is a validated questionnaire [3] containing seven self-report items on four-point scales from 0 to 3 and a maximum total score of 21. Cronbach’s alpha for the current t2 sample = 0.88

Sleep (ISI): The ISI measures sleep issues on a 7-item questionnaire using 4-point likert scales with a maximum score of 28. This measure has been validated in adolescents [4]. Cronbach’s alpha for the current t2 sample = 0.82

Alcohol abuse: The reliable and validated screening interview CAGE [5] was used to identify students with potential problems with alcohol abuse. It consists of four yes/no questions that are weighted equally (each yes answer = 1), and a total score of two or greater is considered clinically significant [6].

Cronbach’s alphas for t1 are reported in a previous article [7].

Results

Whole t2 sample (September to November 2021):

The cut-off for clinically relevant depressive symptoms (i.e. PHQ-9 score ≥11) was exceeded by 61.9% girls, 38.1% boys and 94.1% non-binary students. For anxiety 49.3% girls, 28.8% boys and 70.6% non-binary students had clinically relevant symptoms (i.e. GAD-7 score ≥11). Clinically relevant moderate insomnia (i.e. ISI score ≥15) was reported by 27.5% girls, 16.7% boys and 43.1% students with non-binary gender. The prevalence of suicidal ideation (item 9 of the PHQ-9) within the last 2 weeks was 46.8% in girls, 32.0% in boys and 90.2% in students with non-binary gender identity. The prevalence of alcohol abuse was 16.5%, with no significant gender-difference (*p* = 0.374). A one-way MANOVA showed a statistically significant difference between genders on the combined dependent variables (WHO-5, PHQ-9, GAD-7, ISI), F(8, 2998) = 16.260, *p* < 0.001.

Table S1**.** Measures of psychological health by gender (whole sample) at the beginning of the second semester of reopened schools (14^th^ September 2021 to 14^th^ November 2021).

|  |  | **Total** | **Girls** | **Boys** | **Non-binary** | **Statistics** |
| --- | --- | --- | --- | --- | --- | --- |
| WHO-5 | N | 1505 | 1173 | 281 | 51 |  |
|  | Score, mean (SD) | 36.6 (21.2) | 35.1 (20.5) | 45.2 (22.5) | 23.7 (16.7) | *F*(2,1502)=37.006; *p* < 0.001 |
| PHQ-9 | N | 1505 | 1173 | 281 | 51 |  |
|  | Score, mean (SD) | 12.6 (6.66) | 13.0 (6.41) | 9.56 (6.65) | 18.7 (5.52) | *F*(2,1502)=56.866; *p* < 0.001 |
|  | ≥11, No. (%) | 881 (58.5) | 726 (61.9) | 107 (38.1) | 48 (94.1) | *X^2^*(2,1505) = 80.503; *p* < 0.001 |
|  | Suicidal Ideation, No. (%) | 685 (45.5) | 549 (46.8) | 90 (32.0) | 46 (90.2) | *X^2^*(2,1505) = 62.452; *p* < 0.001 |
| GAD-7 | N | 1505 | 1173 | 281 | 51 |  |
|  | Score, mean (SD) | 10.1 (5.45) | 10.5 (5.33) | 7.57 (5.28) | 13.2 (4.77) | *F*(2,1502)=44.705; *p* < 0.001 |
|  | ≥11, No. (%) | 695 (46.2) | 578 (49.3) | 81 (28.8) | 36 (70.6) | *X^2^*(2,1505) = 50.798; *p* < 0.001 |
| ISI | N | 1505 | 1173 | 281 | 51 |  |
|  | Score, mean (SD) | 10.6 (5.90) | 10.9 (5.83) | 8.73 (5.75) | 13.8 (5.71) | *F*(2,1502)=23.824; *p* < 0.001 |
|  | ≥15, No. (%) | 392 (26.0) | 323 (27.5) | 47 (16.7) | 22 (43.1) | *X^2^*(2,1505) = 21.758; *p* < 0.001 |
| CAGE | ≥2, No. (%) | 248 (16.5) | 189 (16.1) | 47 (16.7) | 12 (23.5) | *X^2^*(2,1505) = 1.969; *p* = 0.374 |

Abbreviations: *p*: *p*-values (2-tailed); N: sample size; SD: standard deviation; ISI: Insomnia Severity Index, GAD-7: Generalized Anxiety Disorder 7 scale; PHQ-9: Patient Health Questionnaire 9 scale; WHO-5: Well-being questionnaire of the World Health Organization (WHO); CAGE: Alcohol abuse screening tool.

The 248 students from t2 not included in the matched analyses could not be suitably matched to students from t1 according to the socio-demographic factors used for matching.

**Qualitative results by gender**

In responding to the question of whether or not they would want support to improve their psychological well-being, a significant difference among gender was observed (*X^2^*(2,1497) = 49.237; *p* < 0.001). While 62.7% of students with non-binary gender and 40.0% of girls affirmed the question of whether they wish for support to improve their psychological wellbeing, the proportion was only 21.1% in boys. Gender differences remained significant when only looking at those participants who exceeded at least one cut-off for clinically relevant depression, anxiety, insomnia or alcohol abuse (*X^2^*(2,1022) = 20.237; *p* < 0.001). Among those students, 50.0% girls, 32.9% boys and 65.3% non-binary students stated that they wish for support to improve their mental health. No gender differences emerged among students scoring below all cut-offs (*X^2^*(2,475) = 3.793; *p* = 0.150).

Of those who answered yes to this question, the proportion of students who gave more detailed information about the type of support did not differ among genders (69.5% of girls, 74.6% of boys, and 78.1% of non-binary students; *X^2^*(2,557) = 1.579; *p* = 0.454).

Analysis of sources of support by gender (Figure S1) revealed a significant difference for “professional help” (*X^2^*(2,393) = 9.801; *p* = 0.007). While 76.0% of students with non-binary gender identity stated that they wish for professional help to improve their mental well-being, this was less often wished for by girls (46.0%) and boys (38.6%). No differences were observed among the other seven categories (all *p*-values ≥0.053).


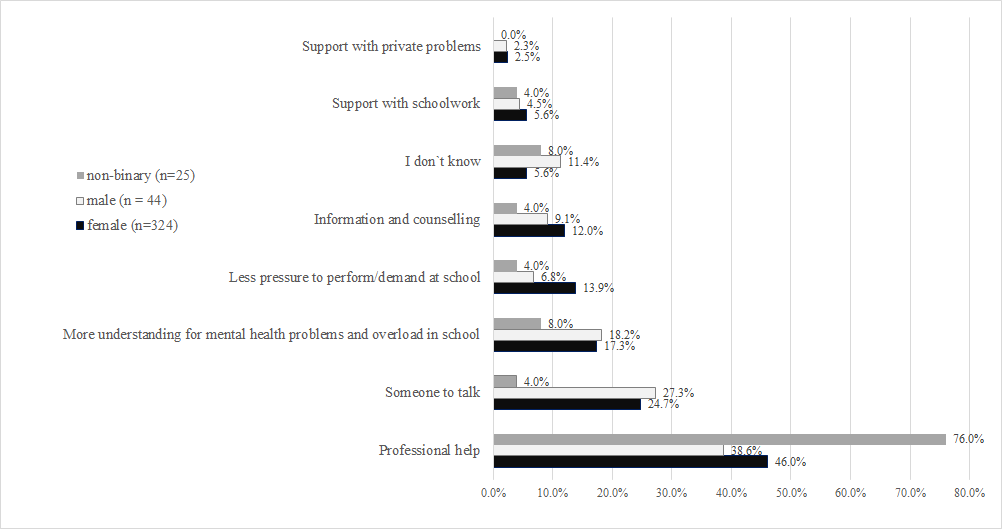

Figure S1. Sources of support requested broken down by gender (girl, boy, non-binary).

Comparisons between the gender groups should be interpreted with caution due to the uneven sample sizes.

**Additional quotes from the qualitative data**

Category – More understanding for mental health problems:

*“Teachers in schools being a little more understanding that we all may have problems we don't talk about and shouldn't expect us to perform perfectly all the time.”* (female student no. 895, age 19)

Category - Someone to talk to:

*“Someone who listens to me and understands me, who doesn't criticise me because I see something differently, who doesn't crush me just because I no longer have the strength to do something.”* (female student no. 947, age 17)

Category - Less pressure to perform/demands at school:

*“Students in general are heavily overburdened. Not only me with my 19 years but also younger ones. Many of my class drink alcohol to forget the stress. I have my school leaving exams soon and in my year you don`t get something for nothing. My class was affected by Corona for more than 2 years and the state doesn't think it's important enough to “help” us with our exams like they did with the students in the last graduating classes. So I would be very happy to get as much help as the classes before. Graduation classes 2021/22 are completely forgotten. THANK YOU!”* (female student no. 2224, age 19)

Category - I don’t know:

*“I have no idea and don't know if my mental state can be improved at all. It's pretty hopeless.”* (female student no. 722, age 16)

*“I honestly don't know what i should talk about, i am stressed.... at least that's how I feel. I can't sleep, I can't get anywhere and I can't do anything about it. But still I can't even sit still, my memory is not so good, I forget very quickly what I wanted and start something new and so it goes all the time. I also have difficulties reading texts or books because I don't understand anything no matter how easily something is described.”* (female student no. 933, age 18)

References

1. Brähler E, Mühlan H, Albani C, Schmidt S (2007) Teststatistische Prüfung und Normierung der deutschen Versionen des EUROHIS-QOL Lebensqualität-Index und des WHO-5 Wohlbefindens-Index. Diagnostica 53:83–96. https://doi.org/10.1026/0012-1924.53.2.83

2. Allgaier AK, Pietsch K, Frühe B, et al (2012) Screening for depression in adolescents: Validity of the patient health questionnaire in pediatric care. Depress Anxiety 29:906–913

3. Löwe B, Decker O, Müller S, et al (2008) Validation and Standardization of the Generalized Anxiety Disorder Screener (GAD-7) in the General Population. Medical Care 46:266–274. https://doi.org/10.1097/MLR.0b013e318160d093

4. Gerber M, Lang C, Lemola S, et al (2016) Validation of the {German} version of the insomnia severity index in adolescents, young adults and adult workers: results from three cross-sectional studies. BMC Psychiatry 16:174. https://doi.org/10.1186/s12888-016-0876-8

5. Ewing JA (1984) Detecting Alcoholism: The CAGE Questionnaire. JAMA 252:1905–1907. https://doi.org/10.1001/JAMA.1984.03350140051025

6. Williams N (2014) The CAGE questionnaire. Occup Med (Chic Ill) 64:473–474. https://doi.org/10.1093/occmed/kqu058

7. Pieh C, Plener PL, Probst T, et al (2021) Assessment of Mental Health of High School Students during Social Distancing and Remote Schooling during the COVID-19 Pandemic in Austria. JAMA Network Open 4:2114866. https://doi.org/10.1001/jamanetworkopen.2021.14866
